# Boerhaave Syndrome in the Intensive Care Unit: A Diagnostic Challenge

**DOI:** 10.7759/cureus.109322

**Published:** 2026-05-20

**Authors:** Melissa Marques, Pedro Mendes de Abreu, Maria João Granjo Redondo, Rita Alves, Nuno Deveza

**Affiliations:** 1 Intensive Care Unit, Coimbra Local Health Unit, Coimbra, PRT

**Keywords:** boerhaave syndrome, endoscopic vacuum therapy, esophageal perforation, intensive care unit, mediastinitis, sepsis, thoracotomy

## Abstract

Spontaneous esophageal rupture (Boerhaave syndrome) is a rare and life-threatening condition associated with high morbidity and mortality, particularly when diagnosis and source control are delayed. We report the case of a 73-year-old man presenting with vomiting, chest pain, and dyspnea. Computed tomography revealed extensive pneumomediastinum, and endoscopy confirmed a distal esophageal perforation with mediastinopleural contamination. The patient developed septic shock requiring intensive care support, including mechanical ventilation and vasopressors. Endoscopic vacuum therapy (EVT) was initially attempted but failed to achieve adequate source control, leading to thoracotomy, pleural debridement, T-tube placement, gastrostomy, and feeding jejunostomy. Pleural cultures grew *Enterococcus faecalis* and *Candida krusei*, prompting escalation of antimicrobial therapy. Despite a prolonged intensive care unit stay and multiple interventions, the patient progressively improved and was discharged for continued recovery. This case highlights the importance of early recognition, multidisciplinary management, and timely escalation from endoscopic to surgical treatment in complex Boerhaave syndrome.

## Introduction

Boerhaave syndrome is a rare form of spontaneous transmural esophageal perforation caused by a sudden increase in intraesophageal pressure, typically following forceful vomiting [1]. Although uncommon, it remains a highly morbid condition due to rapid mediastinal contamination and progression to sepsis when diagnosis or source control is delayed [2]. Spontaneous rupture accounts for approximately 10%-15% of all esophageal perforations and frequently presents with nonspecific symptoms, contributing to diagnostic difficulty and delayed treatment [2,3]. Mortality remains high, particularly in patients presenting with mediastinitis, pleural contamination, or septic shock [3].

Clinical manifestations may include chest pain, dyspnea, vomiting, fever, subcutaneous emphysema, or respiratory failure, often mimicking other thoracic or gastrointestinal emergencies [2,4]. Computed tomography and upper gastrointestinal endoscopy play a central role in confirming the diagnosis and assessing the extent of mediastinal and pleural involvement [4]. Early recognition and multidisciplinary management are essential to improve outcomes and achieve effective source control.

Treatment strategies depend on the location and extent of perforation, time to diagnosis, degree of contamination, and overall clinical status of the patient [5]. Available approaches include conservative management, endoscopic interventions such as stent placement or endoscopic vacuum therapy (EVT), and surgical repair [5,6]. EVT has emerged as a promising minimally invasive alternative, particularly in critically ill patients, with reported success rates exceeding 80% in selected cases [6]. However, persistent leakage, uncontrolled infection, or extensive pleural contamination may still require escalation to surgical treatment [5,6].

We report the case of a 73-year-old man with Boerhaave syndrome complicated by severe mediastinitis, pleural empyema, septic shock, and prolonged intensive care unit admission. Despite initial management with EVT, persistent contamination and clinical deterioration required thoracotomy with pleural debridement, T-tube placement, gastrostomy, and feeding jejunostomy. This case highlights the diagnostic and therapeutic challenges associated with Boerhaave syndrome and underscores the importance of individualized multidisciplinary management and timely escalation from endoscopic to surgical intervention when source control is not achieved.

## Case presentation

A 73-year-old man with a history of chronic alcohol use presented to the emergency department with intractable vomiting, chest pain, and progressive dyspnea following repeated episodes of forceful emesis. Initial evaluation raised suspicion of esophagogastric perforation. Due to rapid clinical deterioration with hemodynamic instability and global respiratory failure, the patient required invasive mechanical ventilation for airway protection, vasopressor support, and transfer to the intensive care unit. Initial laboratory findings demonstrated a marked systemic inflammatory response, consistent with severe mediastinal contamination and sepsis (Table 1).

**Table 1:** Laboratory parameters obtained during the initial evaluation in the intensive care unit, demonstrating marked systemic inflammatory response and organ dysfunction associated with esophageal perforation and mediastinal contamination.

Parameter	Patient Value	Reference Range
Hemoglobin	9.5 g/dL	11.8–15.8 g/dL
White blood cell count (WBC)	10.0 ×10⁹/L	3.60–10.5 ×10⁹/L
Platelets	696 ×10⁹/L	140–385 ×10⁹/L
C-reactive protein (CRP)	23.62 mg/dL	<0.5 mg/dL
Procalcitonin	2.0 ng/mL	<0.1 ng/mL
Lactate	0.76 mmol/L	0.5–2.0 mmol/L
Creatinine	0.45 mg/dL	0.7–1.18 mg/dL
Sodium	138 mmol/L	136–146 mmol/L
Potassium	4.4 mmol/L	3.5–5.1 mmol/L
Aspartate aminotransferase (AST)	75 U/L	<35 U/L
Alanine transaminase (ALT)	43 U/L	<45 U/L

Empirical antimicrobial therapy with meropenem and fluconazole was initiated. Computed tomography revealed extensive pneumomediastinum with distal esophageal thickening (Figure 1). Upper gastrointestinal endoscopy confirmed a distal esophageal perforation with extensive mediastinopleural contamination (Figure 2). EVT was initiated using an endoluminal sponge system.

**Figure 1:**
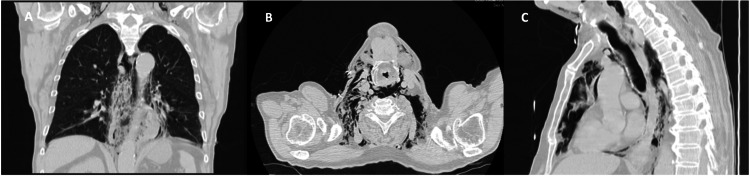
Chest computed tomography showing extensive pneumomediastinum with distal esophageal thickening, consistent with esophageal perforation. (A) Coronal view; (B) Axial view; (C) Sagittal view

**Figure 2:**
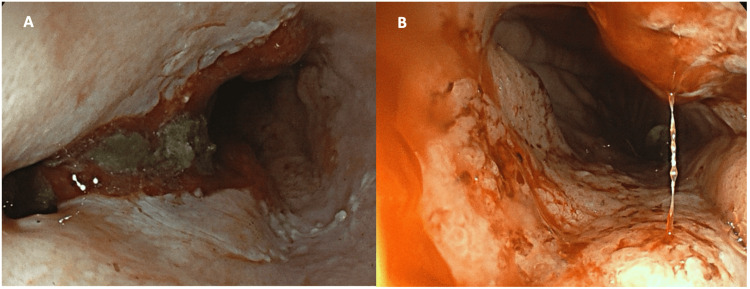
Upper gastrointestinal endoscopy showing distal esophageal perforation (A) and subsequent resolution with surrounding granulation tissue (B).

Despite repeated EVT exchanges, the patient developed persistent pleural contamination with food debris and purulent material, recurrent infectious complications, and prolonged ventilatory dependence. During the intensive care unit stay, pleural fluid cultures demonstrated polymicrobial infection, including *Enterococcus faecalis*, *Candida krusei*, *Staphylococcus epidermidis*, and *Proteus mirabilis*. Antimicrobial susceptibility testing showed multisensitive *Enterococcus faecalis*, methicillin-resistant *Staphylococcus epidermidis* susceptible only to vancomycin, and extended-spectrum beta-lactamase-negative *Proteus mirabilis*. Blood and respiratory cultures remained negative. These findings prompted escalation of antimicrobial therapy from fluconazole to anidulafungin and addition of vancomycin according to microbiological results and clinical evolution.

Due to persistent contamination and lack of clinical improvement, the patient underwent left thoracotomy and laparotomy on hospital day 14, including pleural debridement, T-tube placement, gastrostomy for drainage, and feeding jejunostomy. Subsequent endoscopic reassessment demonstrated progressive granulation tissue formation and partial resolution of the esophageal defect (Figure 2).

The patient required prolonged intensive care support, including difficult ventilatory weaning, nutritional support, repeated endoscopic procedures, and multidisciplinary management involving gastroenterology, thoracic surgery, general surgery, infectious diseases, and intensive care teams. After a prolonged intensive care unit stay of 32 days, the patient progressively improved and was transferred to a step-down care unit for continued recovery.

## Discussion

Spontaneous esophageal rupture, known as Boerhaave syndrome, is a rare but life-threatening condition resulting from a sudden increase in intraesophageal pressure, typically following forceful vomiting [2]. It most commonly affects the distal esophagus and leads to rapid contamination of the mediastinum and pleural cavity, with potential progression to severe sepsis and multiorgan failure if not promptly treated [1]. Mortality remains high, particularly when diagnosis or source control is delayed beyond the first 24 hours [3].

Clinical presentation is frequently nonspecific and may mimic other thoracic or gastrointestinal emergencies, contributing to delayed diagnosis. Although classically associated with Meckler’s triad of vomiting, chest pain, and subcutaneous emphysema, this combination is present in only a minority of patients [4]. In the present case, the initial presentation with vomiting, chest pain, and dyspnea rapidly progressed to respiratory failure and septic shock requiring intensive care admission and invasive mechanical ventilation.

Computed tomography plays a central role in early diagnosis, particularly through the identification of pneumomediastinum, pleural effusion, mediastinal fluid collections, and esophageal wall abnormalities [5]. Upper gastrointestinal endoscopy allows direct visualization of the perforation and assessment of contamination extent, although its use should be individualized given the theoretical risk of worsening the defect [5]. In our patient, early endoscopic evaluation was essential for diagnosis and subsequent therapeutic planning.

Management of Boerhaave syndrome remains challenging and requires an individualized multidisciplinary approach involving gastroenterology, thoracic surgery, general surgery, infectious diseases, and intensive care teams [3]. Treatment options range from conservative management to endoscopic and surgical interventions, depending on the timing of diagnosis, degree of contamination, and overall clinical stability [3,5].

EVT has emerged as an increasingly used minimally invasive strategy for esophageal perforations, with reported success rates exceeding 80% in selected patients [6]. However, treatment failure may occur in cases with extensive mediastinal or pleural contamination, persistent leakage, necrotic tissue, or uncontrolled infection [6]. In the present case, despite repeated EVT exchanges, the patient developed progressive pleural contamination with food debris and purulent material, persistent infection, and prolonged ventilatory dependence, ultimately requiring surgical intervention for definitive source control.

Similar reports describe delayed diagnosis, severe pleural contamination, prolonged intensive care admission, and high mortality, frequently requiring combined endoscopic and surgical management [3,5]. In our patient, thoracotomy with pleural debridement and T-tube placement, combined with gastrostomy and feeding jejunostomy, enabled effective drainage, nutritional support, and progressive healing of the perforation.

Infectious complications were particularly relevant during the clinical course. Pleural cultures demonstrated polymicrobial contamination, including *Enterococcus faecalis*, *Candida krusei*, *Staphylococcus epidermidis*, and *Proteus mirabilis*, requiring broad-spectrum antibacterial and antifungal therapy. Fungal isolation in upper gastrointestinal perforation is associated with increased morbidity and may reflect extensive contamination of the mediastinal and pleural spaces [7]. This case reinforces the importance of repeated microbiological assessment and individualized antimicrobial escalation in critically ill patients with persistent infection. 

Despite prolonged intensive care admission, difficult ventilatory weaning, repeated endoscopic procedures, and major surgical intervention, the patient achieved a favorable outcome. This case highlights the importance of early recognition, close multidisciplinary collaboration, and timely escalation from endoscopic to surgical management when adequate control of mediastinal and pleural contamination cannot be achieved.

## Conclusions

This case illustrates the diagnostic and therapeutic complexity of Boerhaave syndrome in critically ill patients. Progressive mediastinal and pleural contamination may persist despite endoscopic treatment, requiring repeated reassessment and escalation to surgical source control. Persistent sepsis, prolonged ventilatory dependence, and recurrent pleuropulmonary infection should prompt ongoing reassessment for uncontrolled mediastinal or pleural contamination. A multidisciplinary approach involving intensive care, gastroenterology, surgery, and infectious diseases teams was fundamental for the favorable outcome achieved in this patient.
